# The Reproducibility of Ultrasonographic Findings of Rectosigmoid Endometriosis Among Examiners With Different Level of Expertise

**DOI:** 10.1002/jum.15717

**Published:** 2021-04-10

**Authors:** Stefano Guerriero, MariaAngela Pascual, Silvia Ajossa, Manuela Neri, Monica Pilloni, Betlem Graupera, Ignacio Rodriguez, Juan Luis Alcazar

**Affiliations:** ^1^ Centro Integrato di Procreazione Medicalmente Assistita (PMA) e Diagnostica Ostetrico‐Ginecologica Policlinico Universitario Duilio Casula, University of Cagliari Cagliari; ^2^ Department of Obstetrics, Gynecology, and Reproduction Hospital Universitari Dexeus Barcelona Spain; ^3^ Department of Obstetrics and Gynecology University of Cagliari, Policlinico Universitario Duilio Casula Cagliari Italy; ^4^ Departamento de Obstetricia, Ginecología y Reproducción Hospital Universitario Quirón Dexeus, Unidad Epidemiología y Estadística Barcelona Spain; ^5^ Department of Obstetrics and Gynecology, Clínica Universidad de Navarra, School of Medicine University of Navarra Pamplona Spain

**Keywords:** deep endometriosis, rectosigmoid endometriosis, reproducibility, three‐dimensional ultrasonography (3D), ultrasound

## Abstract

**Objective:**

To analyze the reproducibility of ultrasonographic (US) findings of rectosigmoid endometriosis among examiners with different level of expertise using stored three‐dimensional (3D) volumes of the posterior compartment of the pelvis as a part of SANABA (Sardinia‐Navarra‐Barcelona) collaborative study.

**Materials and methods:**

Six examiners in 3 academic Department of Obstetrics and Gynecology, with different levels of experience and blinded to each other, evaluated 60 stored 3D volumes from the posterior compartment of the pelvis and looked for the presence or absence of features of rectosigmoid endometriotic lesions defined as an irregular hypoechoic nodule with or without hypoechoic foci at the level of the muscularis propria of the anterior wall rectum sigma. Multiplanar view and virtual navigation were used. All examiners had to assess the 3D volume of posterior compartment of the pelvis and classify it as present or absent disease. To analyze intra‐observer and the inter‐observer agreements, each examiner performed the assessment twice with a 2‐week interval between the first and second assessments. Reproducibility was assessed by calculating the weighted Kappa index.

**Results:**

Intra‐observer reproducibility was moderate to very good for all observers (Kappa index ranging from 0.49 to 0.96) associated with a good diagnostic accuracy of each reader. Inter‐observer reproducibility was fair to very good (Kappa index range: 0.21–0.87).

**Conclusions:**

The typical US sign of rectosigmoid endometriosis is reasonably recognizable to observers with different level of expertise when assessed in stored 3D volumes.

**Abbreviations**
3D: three dimensional
NPV: negative predictive value
PPV: positive predictive value
SANABA: Sardinia‐Navarra‐Barcelona
US: ultrasonographic

The use of transvaginal ultrasound is well established in the diagnosis of rectosigmoid endometriosis with high accuracy when studies are performed by expert operators as demonstrated by several meta‐analyses published in the last years.^1^, ^2^, ^3^, ^4^, ^5^, ^6^ To improve the diffusion of this diagnostic method, it is necessary to demonstrate that the ultrasonographic findings related with the presence of this disease are reproducible also in the hand of less expert operators. The few studies about reproducibility present in the literature are related to expert operators (usually no more than 2) of the same center.^8^, ^9^, ^10^

To the best of our knowledge, no study has reported if less expert operators can accurately replicate the results of experts. The aim of the present multicenter study was to evaluate the reproducibility of ultrasonographic (US) findings of rectosigmoid endometriosis among examiners with different level of expertise in different centers using stored 3D volumes of the posterior compartment of the pelvis as a part of SANABA (Sardinia‐Navarra‐Barcelona) collaborative study.

## Materials and Methods

All the patients, submitted to ultrasonography for pelvic pain and/or infertility, whose volumes were included in the present study signed an informed consent for the first time they are examined, consenting their anonymized data can be used in clinical research. Six examiners in 3 academic Department of Obstetrics and Gynecology, with different levels of experience in gynecological ultrasound and blinded to each other, evaluated 60 stored three‐dimensional (3D) volumes from the posterior compartment of the pelvis. Due to the retrospective and observational design and anonymization, Institutional Review Board approval was waived. The study was performed between September 2019 and January 2020. All volumes were acquired using a Voluson E8 (GE Healthcare, Milwaukee, IL) equipped with a 5–9 MHz endovaginal probe, after a complete 2D transvaginal ultrasound assessment of the pelvis. These volumes were chosen randomly by 1 of the authors (M. A. P.) from the central database, which contains more than 300. All selected 3D volumes had been recorded by the author, who routinely performed 3D acquisition in all patients with suspected endometriosis. Inclusion criteria were based on the fact that the posterior compartment was entirely or almost entirely included in the 3D volume including the posterior part of the uterus using the maximum angle of insonation.

**Figure 1:**
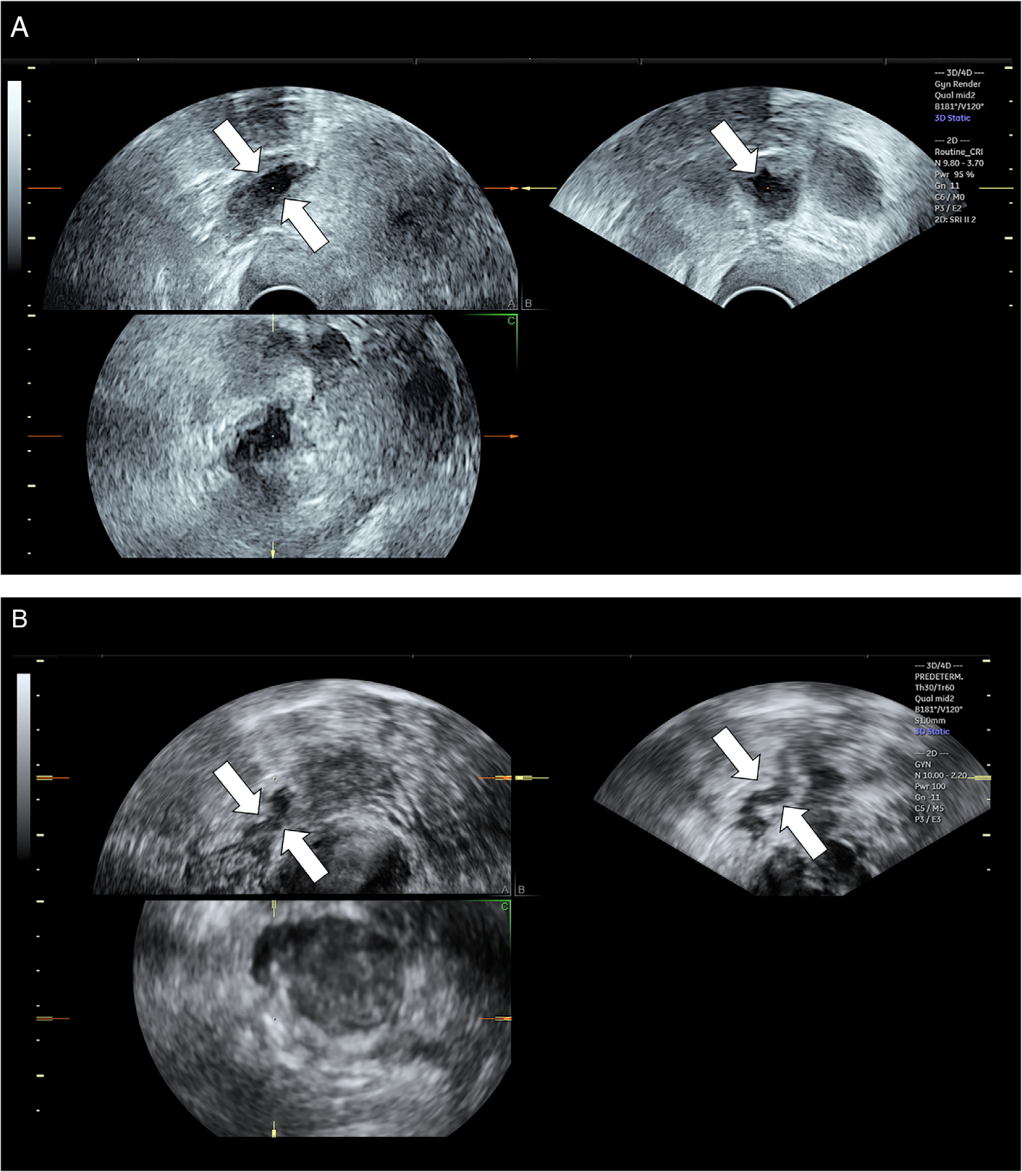
**A** and **B**. Two (**A** and **B**) irregular hypoechoic nodules (see arrows) at the level of the muscularis propria of the anterior wall rectum sigma considered characteristic findings of endometriotic nodule of recto‐sigma.

Definitive surgical diagnosis was available for all the cases. The 6 examiners who assessed the 3D volumes were blinded to surgical results and patient data. Three examiners (Seniors 1, 2, and 3, one for each center) were considered experts, being gynecologists specially devoted to gynecological ultrasound with over 15 years of experience. Three examiners (Juniors 1, 2, and 3, one for each center) were trainees in obstetrics and gynecology, with less than 2 years of experience in gynecological ultrasound. In particular, all the residents had basic training in real‐time transvaginal ultrasound. Additionally, all were trained in using 4‐D View software (4D View, GE Healthcare, Zipf, Austria) and all performed a visual training about how rectosigmoid lesions usually appear by means of a theoretical presentation with example cases. Each examiner had to assess the 3D volume using the dedicated software (4D View) and look for the presence or absence of features of rectosigmoid endometriotic lesions defined as an irregular hypoechoic nodule with or without hypoechoic foci at the level of the muscularis propria of the anterior wall rectum sigma^11^ (Figure 1). All examiners had to assess the 3D volume of the posterior compartment of the pelvis and determine if a rectosigmoid endometriotic lesion was present or not. Assessment was performed using the multiplanar view and virtual navigation through the posterior compartment.

To analyze intra‐observer agreement, each examiner performed the assessment twice with a 2‐week interval between the first and second assessments. The second assessment from each examiner was usually analyze the inter‐observer agreement. Reproducibility was assessed by calculating the weighted Kappa index.^12^ A Kappa value of 0.20 or less indicates poor agreement; 0.21–0.40 indicate fair agreement; 0.41–0.60 indicate moderate agreement; 0.61–0.80 indicate good agreement; and 0.81–1.00 indicate very good agreement.^12^ Using the definitive surgical diagnosis as the reference standard, sensitivity and specificity (with 95% CI) for the echo features suggestive of the presence of rectosigmoid endometriosis were calculated to compare the diagnostic performance of operators with varying degrees of experience.

## Results

Among the 60 stored 3D volumes, 17 contained a rectosigmoid nodule confirmed by surgery (28.33%). Intra‐observer reproducibility was moderate to very good for all observers (Kappa index ranging from 0.49 to 0.96; Table 1) with a substantial percentage of agreement ranging from 98 to 75%. Lower Kappa values were present in the majority of less experienced operators but with an overlap of 95% confidence intervals. The diagnostic accuracy of each reader was higher in the experts in comparison with less experienced operators (0.94–0.82 versus 0.76) but with an overlap of 95% confidence intervals (Table 1). The values of specificity were good ranging from 0.95 to 0.63 with a sensitivity ranging from 0.94 to 0.76.

**Table 1:** Intra‐observer Reproducibility and Accuracy for Observers With Different Levels of Expertise

Observer	Kappa Index^a^	Sensitivity^a^	Specificity^a^	PPV^a^	NPV^a^	Accuracy^a^	Percentage of Agreement^a^
Senior 1	0.96 (0.71–1.21)	0.94 (0.71, 1.00)	0.95 (0.84, 0.99)	0.89 (0.65–0.99)	0.98 (0.87–1.00)	0.95 (0.86–0.99)	98.3% (91.1–100%)
Senior 2	0.83 (0.58–1.09)	0.82 0.82 (0.57, 0.96)	0.70 0.70 (0.54, 0.83)	0.52 (0.32, 0.71)	0.91 (0.76, 0.98)	0.73 (0.60–0.84)	91.7% (81.6–97.2%)
Senior 3	0.78 (0.52–1.03)	0.82 0.82 (0.57, 0.96)	0.84 0.84 (0.69, 0.93)	0.67 (0.43, 0.85)	0.92 (0.79, 0.98)	0.83 (0.71–0.92)	90% (79.5–96.2%)
Junior 1	0.95 (0.7–1.21)	0.76 0.76 (0.50, 0.93)	0.95 0.95 (0.84, 0.99)	0.87 (0.60, 0.98)	0.91 (0.79, 0.98)	0.9 (0.79–0.96)	98.3% (91.1–100%)
Junior 2	0.66 (0.41–0.92)	0.76 0.76 (0.50, 0.93)	0.63 0.63 (0.47, 0.77)	0.45 (0.26, 0.64)	0.87 (0.70, 0.96)	0.67 (0.53–0.78)	83.3% (71.5–91.7%)
Junior 3	0.49 (0.24–0.74)	0.76 0.76 (0.50, 0.93)	0.70 0.70 (0.54, 0.83)	0.50 (0.30, 0.70)	0.88 (0.73, 0.97)	0.72 (0.58–0.82)	75% (62.1–85.3%)

PPV, positive predictive value; NPV, negative predictive value.

^a^ 95% confidence intervals in parentheses.

Inter‐observer reproducibility was fair to very good (Kappa index range: 0.21–0.87) (Table 2) without differences related to the experience of operators. The percentage of agreement ranging from 61.7 to 95% did not show differences based on the degree of experience of the operators.

**Table 2:** Inter‐observer Agreement Between Observers, Expressed as Kappa Index and Percentage of Agreement

	Senior 2^a^	Senior 3^a^	Junior 1^a^	Junior 2^a^	Junior 3^a^
Senior 1	0.49 (0.25–0.72) 75% (62.1–85.3%)	0.6 (0.35–0.86) 85% (73.4–92.9%)	0.87 (0.62–1.12) 95% (86.1–99%)	0.5 (0.25–0.74) 68.3% (55–79.7%)	0.35 (0.11–0.6) 73.3% (60.3–83.9%)
Senior 2		0.21 (0.03–0.45) 66.7% (53.3–78.3%)	0.38 (0.16–0.6) 70% (56.8–81.2%)	0.63 (0.38–0.88) 66.7% (53.3–78.3%)	0.27 (0.02–0.52) 65% (51.6–76.9%)
Senior 3			0.54 (0.3–0.79) 83.3% (71.5–91.7%)	0.36 (0.11–0.61) 70% (56.8–81.2%)	0.43 (0.18–0.68) 75% (62.1–85.3%)
Junior 1				0.38 (0.15–0.61) 66.7% (53.3–78.3%)	0.23 (0–0.46) 75% (62.1–85.3%)
Junior 2					0.25 (0.01–0.5) 61.7% (48.2–73.9%)

^a^ 95% confidence intervals in parentheses.

## Discussion

The typical ultrasonographic sign of rectosigmoid endometriosis is reasonably recognizable to observers with different level of expertise when assessed in stored 3D volumes in our multicenter study. Lower Kappa values, as expected, were present in the majority of less experienced operators in terms of intra‐observer reproducibility but these operators always remained in the range of moderate or good agreement with all values higher than 0.40 (Table 1). Regarding inter‐observed reproducibility, the observed Kappa values indicate a fair to very good (Table 2) without differences attributable to the experience of operators.

Regarding previous studies about reproducibility of ultrasound in the diagnosis of endometriosis, 2 were performed using real‐time ultrasound by expert operators .^8^, ^10^Two other studies used an offline approach to avoid discomfort to patients with such a usually painful disease. Reid et al^7^ used videos in 30 women to evaluate only the siding sign and Egekvist et al^9^ used three‐dimensional volumes and expert operators to evaluate intra‐observer and inter‐observer variability of measurement of endometriotic nodules.

The present study justifies the use of transvaginal ultrasonography in less expert operators to diagnose endometriotic disease in the posterior compartment but has some limitations, since our analysis, as stated previously, was performed using stored 3D volumes instead of real‐time ultrasound. Probably the results of the present study would be even better if real‐time clips of the posterior compartment had included for, to look for free sliding of structures with gentle pressure of the probe versus adherence of structures due to scarring. Nevertheless, there is some evidence that, for example, in the diagnosis of adnexal masses, the analysis of 3D stored volumes may render similar diagnostic performance to real‐time ultrasound^13^ and that assessment of tumor features is reproducible among different observers analyzing the same stored 3D volume.^14^, ^15^ Kappa values found in the present study were higher than values obtained for intra‐observer reproducibility of the now well‐established IOTA simple ultrasound rules for classifying adnexal masses as benign or malignant.^16^

One of the main criticisms regarding the use of transvaginal ultrasonography is the so‐called operator dependency. Notwithstanding the fact that magnetic resonance accuracy is also related to the experience of the radiologist in the field of assessment of the pelvis,^17^ it has recently been suggested that an examiner who is familiar with transvaginal ultrasonography can achieve proficiency in the diagnosis of deep infiltrating endometriosis after performing about 40 examinations.^18^ Other authors^19^ also showed that a sonographer trained in general gynecologic ultrasonography, who has invested time to learn transvaginal ultrasonography for deep infiltrating endometriosis mapping, can achieve proficiency for diagnosing the major types of endometriotic lesions after examining less than 50 patients. Regarding the use of 3D ultrasound as educational tools to improve the learning curve of less expert operators, Guerriero et al^20^ found that the combined use of real‐time transvaginal ultrasonography and offline 3D volumes virtual navigation was helpful to improve training, in a short period of time (2 weeks), for ultrasound assessment of deep endometriosis. In his proposed learning program, with concentration of cases during the training period, competence for diagnosis in the rectosigmoid locations was reached after only 39 evaluations. In this study, the accuracy for each trainee was high, ranging from 80 to 94% after training. These results are partially in agreement with the present study where we demonstrated a sensitivity of 76% and a specificity ranging from 95 to 63%, respectively, for less expert operators. Although we observed a wide range of accuracies between less expert operators, all had similar level of training in ultrasound and software use. Probably in the future more specific protocols have to be used to reduce these differences. For example, performing a combined use of real‐time transvaginal ultrasonography and offline 3D volumes virtual navigation previously demonstrated as efficient to reduce the learning curve.^20^

A recent metanalysis^6^ confirmed previous papers,^1^, ^2^, ^3^ showing that the pooled sensitivity and specificity of magnetic resonance for rectosigmoid location were 88 and 90%, and the values of transvaginal ultrasonography were 90 and 96%, respectively. The present study, although with the provision of having used 3D volumes and not in real time, showed good results in terms of accuracy also in less experienced operators with accuracy for experts ranging from 0.95 to 0.73 and for less experts ranging from 0.9 to 0.67. Therefore, the suggestion that magnetic resonance has to be considered as a valuable approach in settings where skilled sonographers are not available^5^ can become obsolete because the aim in the next years will be to extend the use of a structured transvaginal ultrasonographic evaluation (according to IDEA consensus)^21^, ^22^, ^23^, ^24^, ^25^, ^26^ in every Department of Obstetrics and Gynecology for all patients with suspicion of endometriosis since the reproducibility of ultrasonographic findings is acceptable and the learning curve not as long as previously suggested.^18^, ^19^, ^20^

## Conclusion

The typical US sign of rectosigmoid endometriosis is reasonably recognizable to observers with different level of expertise when assessed in stored 3D volumes.
